# Follicular flushing increases the number of oocytes retrieved in poor ovarian responders undergoing in vitro fertilization: a retrospective cohort study

**DOI:** 10.1186/s12905-018-0681-2

**Published:** 2018-11-16

**Authors:** Yu Xiao, Yong Wang, Min Wang, Kai Liu

**Affiliations:** Reproductive Medical Center, The International Peace Maternity and Child Health Hospital, School of Medicine, Shanghai Jiaotong University, 910 Hengshan Road, Shanghai, 200030 China

**Keywords:** Follicular flushing, Oocyte retrieval, In vitro fertilization, Poor ovarian response

## Abstract

**Background:**

To investigate the impact of follicular flushing on the number of oocytes retrieved and embryo quality and to determine the optimal number of flushings for poor ovarian responders (PORs) undergoing in vitro fertilization (IVF).

**Methods:**

This retrospective study included 291 IVF cycles in 224 patients who were PORs and had no more than three dominant follicles on retrieval day. During oocyte retrieval, follicular fluid was aspirated and examined for an oocyte. If no oocyte was identified, follicular flushing was repeated until an oocyte was retrieved or up to a maximum of nine times.

**Results:**

The mean number of oocytes retrieved by aspiration and subsequent flushes was significantly higher than the number retrieved from the initial aspirate (1.73 ± 0.96 VS. 1.23 ± 1.00, *P* = 0.000). The total recovery rate was 83.7% (503/601), which was significantly higher than the 59.6% recovery rate for direct aspiration (*P* = 0.000). Before the 4th follicular flushing, the cumulative recovery rate increased significantly as flushing was repeated, but after the 4th flushing, the ascending trend was mitigated; and the risk ratio of recovering fewer oocytes after 4 flushes compared with after 9 flushes was 0.765 (95%CI, 0.570–1.026, *P* = 0.074). Significant differences were not observed in maturation rate, fertilization rate, cleavage rate or high-quality embryo rate (*P* > 0.05).

**Conclusions:**

Follicular flushing may increase the number of oocytes retrieved and does not have adverse effects on oocyte or embryo quality in PORs undergoing IVF. Four times may be an optimal number of follicular flushings.

## Background

Oocyte retrieval is a critical step in assisted reproductive technology (ART). It was first performed via laparoscopy, which was complicated and of low efficiency. Ultrasound-guided transvaginal oocyte retrieval was safer and more effective; it is currently the standard operation for in vitro fertilization (IVF) treatment [1, 2]. Theoretically, oocyte retention is possible after the initial aspirate due to abnormal development of the follicle or oocyte and human technical factors, and such retention could be overcome by recurrent follicular flushing [3, 4]. Follicular flushing is believed to maximize the number of oocytes retrieved and subsequently improve the pregnancy rate of IVF [5, 6].

Poor ovarian response (POR) is observed in approximately 9% of patients undergoing ovarian stimulation [7]. Patients with POR have a low response to gonadotrophins, which results in the recovery of few oocytes, a reduced number of valid embryos for transfer, and relatively low pregnancy rates in IVF treatment [8]. Various protocols have been suggested to improve the outcomes of POR patients [9], and increasing the number of oocytes may be one of them. According to the theory of Sunkara S, et al. [10] the live birth rate benefits when larger numbers of oocytes (up to 15) are recovered. Therefore, follicular flushing may be a plausible aspect of IVF treatment for POR patients.

Several observational and non-randomized controlled studies have suggested that flushing results in a higher number of retrieved oocytes [3, 4, 11, 12]. However, in the last decade, additional randomized controlled trials (RCTs) [13–15] have failed to demonstrate that follicular flushing could improve the number of retrieved oocytes and mature oocytes and pregnancy outcomes when compared with direct aspiration. In addition, flushing was obviously associated with an increase in the duration of the retrieval procedure and the use of anesthetic agents [16]. Currently, clinicians have almost reached a consensus that in unselected patients or normal ovarian responders, follicular flushing has very limited benefit and should not be encouraged in routine treatment, though in other groups of patients, such as PORs and those undergoing natural cycle or minimal stimulation IVF, evidence is lacking [17–19].

Hence, we conducted this study to evaluate the potential efficacy of follicular flushing for oocyte retrieval and embryo quality in POR patients undergoing IVF treatment.

## Methods

We performed a retrospective study of the data from patients who underwent IVF at our reproductive center between July 2016 and November 2017. In the routine procedure of our center, follicles with a diameter no more than 10 mm were not punctured. Follicular flushing was performed in all unselected women with no more than 5 follicles with a diameter above 12 mm. The information of repeated flushes was recorded only in patients presenting no more than 3 follicles > 14 mm. The follicles with a diameter from 12 to 14 mm were considered to be not dominant. They were flushed, but not documented.

When the study was conducted, we extracted the information of follicular flushing from the database, from which women aged 36 to 45 years and diagnosed as PORs were included. Other inclusion criteria were as follows: (1) experiencing infertility caused by factors including advanced age, fallopian tube factors, oligospermia, and asthenospermia; (2) undergoing ovarian stimulation protocols, including minimal protocol and natural cycle; (3) receiving urinary or recombinant human chorionic gonadotrophin (u−/r-HCG) to induce oocyte maturation; (4) presenting no more than 3 follicles with a diameter from 16 to 22 mm and < 5 follicles more than 12 mm at oocyte retrieval; and (5) submitted to a maximum of nine follicular flushings. The exclusion criteria were (1) infertility caused by severe oligoasthenospermia and azoospermatism; (2) fertilization by intracytoplasmic sperm injection; and (3) cycles in which no oocyte was retrieved but no more than 9 flushings were performed. The study included 224 patients and 291 IVF cycles.

### Ovarian stimulation

(1) Minimal protocol: On the 2nd to 5th day of the menstrual cycle, the patients received a 5-day administration of clomiphene (Fertilan, CODAL, Cyprus) 50–100 mg/d or letrozole (Furui, HengRui, China) 2.5–5 mg/d, and from then on, 75–150 IU of human menopausal gonadotropin (Menotrophins for Injection, Livzon, China) to induce ovulation. The dosages were adjusted based on ultrasonography monitoring of follicular size and serum sex hormone levels. (2) Natural cycle: no medicine was used, and the follicle developed spontaneously. When the majority of the follicles were larger than 16–18 mm in diameter, 6000 IU of u-HCG (Livzon, China) or 250 μg of r-HCG (Ovidrel, Merck-Serono, Switzerland) was used to induce oocyte maturation.

### Oocyte retrieval

Oocyte retrieval was performed 34–36 h after HCG injection by transvaginal ultrasonography-guided aspiration. All retrievals were performed by one of two experienced physicians. We used a 17-gauge double-lumen puncture needle (K-OPSD-1735-B-L, COOK, Australia) connected to a vacuum pump (K-MAR-5200, COOK, Australia) with pressure of approximately 140 mmHg. Follicular fluid was aspirated and placed in 14-ml test tubes (Falcon, USA). To eliminate the “dead space” effect of the double-lumen needle, 1.8 ml buffered medium (PBS, Irvine Scientific, USA) was injected into the follicle and aspirated again. The two portions of aspirated fluid were considered the direct aspirated follicular fluid and were examined by embryologists for an oocyte-corona-cumulus complex (OCCC). Subsequent flushing was performed if an OCCC was not identified. At each flushing, the follicles were washed with a fixed volume of 3 ml buffered medium, and the flushing fluid was rechecked for an OCCC. Flushing was repeated until the OCCC was retrieved or a maximum of 9 times. While an oocyte was retrieved, the operator announced the number of flushings, and an assistant had it documented. At the same time, the embryologist who had picked up the oocyte assigned it a number and tagged it for further observation.

These procedures, as our standard routine, were performed in all unselected women with no more than 3 follicles > 14 mm. When this study was conducted, the data of the oocytes retrieved from the follicles < 16 mm were excluded, and only the data of PORs were taken into analysis.

### IVF and embryo culture

On the day of oocyte retrieval, the maturity of the OCCCs was classified using the method reported by Veeck L, et al. [20] and conventional IVF was conducted. Fertilization was observed on the 1st day after oocyte retrieval, and the appearance of two pronuclei indicated normal fertilization. On the 3rd day, the cleavage and development of the embryos were observed, and high-quality embryos were defined as embryos that had normal cleavage, no fewer than six blastomeres, a regular or slightly heterogeneous diameter, and less than 20% fragments.

### Statistical analysis

The basic data are expressed as the mean ± SD. The mean number of oocytes retrieved from the aspiration alone and aspiration+flushing groups were compared by using Student’s t-test. To evaluate the effect of the number of flushings on oocyte retrieval, we used binary logistic regression with the number of flushings as the categorical covariate and aspiration alone (no flushing) or 9 flushes as the contrasts to calculate the risk ratios (RRs) and 95% confidence intervals (CIs) of each accumulative flushing time. Pearson’s chi-square test and Fisher’s exact test were used to compare the proportions of oocytes and embryos between groups. A *P*-value of ≤0.05 was considered statistically significant. We used SPSS Statistics, version 23.0 ((IBM Corporation, Armonk, NY, USA) for statistical analyses and Excel 2016 for creating figures.

## Results

A total of 291 cycles of IVF with oocyte retrieval were included. The mean age was 39.32 ± 2.55 years, and other clinical characteristics of the patients are summarized in Table 1. The mean number of follicles aspirated per IVF cycle was 2.07 ± 0.85. The mean number of oocytes retrieved by aspiration and all subsequent flushes was significantly higher than the number retrieved from the initial aspirate (1.73 ± 0.96 VS. 1.23 ± 1.00, *P* = 0.000).

**Table 1:** Demographics, treatment characteristics, and oocyte and embryo outcomes of trial participants (*n* = 291)

	Poor ovarian responders
	N (%)
Primary infertility	102(35.1%)
Secondary infertility	189(64.9%)
	Mean ± SD
Age (years)	39.32 ± 2.55
Duration of infertility (years)	5.44 ± 3.65
Peak estradiol (pmol/L)	2665.11 ± 2401.38
Follicles aspirated	2.07 ± 0.85
Oocytes retrieved	
Initial aspirate	1.23 ± 1.00 ^a^
Aspiration and all flushes	1.73 ± 0.96 ^a^
Mature oocytes	1.54 ± 0.94
Number of fertilizations	1.46 ± 0.96
Number of cleavages	1.43 ± 0.95
Number of high-quality embryos	1.03 ± 0.87

^a^ Differences in the number of oocytes retrieved from the initial aspirate group and the aspiration+all flushes group were significant (Student’s t-test, *P* = 0.000)

Table 2 shows the recovery rates of initial aspirate, and the total recovery rates in different categories. There was no significant difference between two trigger drugs, or between two physicians (*P* > 0.05). Comparing with the natural cycle, the minimal stimulation protocol had a slight higher initial recovery rate (60.5% VS. 46.2%, *P* = 0.078), and a significant higher total recovery rate (84.5% VS. 71.8%, *P* = 0.038).

**Table 2:** Recovery rates in different categories

	N (%)	Recovery rate, n (%)			
		Initial aspirate	*P*-value	Aspirate & all flushes	*P*-value
Ovarian stimulation protocol			0.078		0.038
Minimal stimulation	257(88.3%)	340/562 (60.5%)		475/562 (84.5%)	
Natural cycle	34(11.7%)	18/39 (46.2%)		28/39 (71.8%)	
Drug used for trigger			0.105		0.121
u-HCG	79(27.1%)	89/164 (54.3%)		131/164 (79.9%)	
r-HCG	212(72.9%)	269/437 (61.6%)		372/437 (85.1%)	
Physician performing the retrieval			0.528		0.556
Physician A	133 (45.7%)	163/280 (58.2%)		237/280 (84.6%)	
Physician B	158 (54.3%)	195/321 (60.7%)		266/321 (82.9%)	

*u-HCG* urinary human chorionic gonadotrophin, *r-HCG* recombinant human chorionic gonadotrophin

**Fig. 1:**
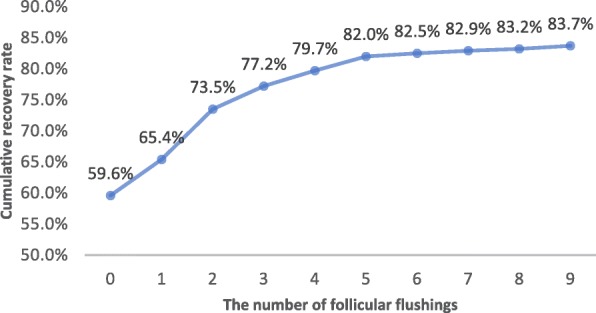
The number of follicular flushings and cumulative recovery rates. Up to the 4th flushing, each flushing contributed to an obvious ascending tendency in the cumulative recovery rate; however, with 5 or more flushings, the upward trend was greatly mitigated

Of the total of 601 punctured follicles, 503 OCCCs were obtained, yielding a recovery rate of 83.7%. Table 3 shows the proportion of oocytes recovered and the cumulative recovery rate for the initial aspirate and each flushing. Only 59.6% of the oocytes were collected with direct aspiration, and the proportion of all oocytes retrieved was 71.2%. When subsequent flushes were performed, more oocytes were recovered. Even with the 9th flushing, there was still a small incidence of oocyte recovery (0.6% in proportion). Up to the 4th flushing, each flushing contributed to an obvious ascending tendency in the cumulative recovery rate; however, with 5 or more flushings, the upward trend was greatly mitigated (Fig. 1).

**Table 3:** Proportion of recovered oocytes and cumulative recovery rate for each flushing

	The number of follicular flushings										Total
	0	1	2	3	4	5	6	7	8	9	
Oocytes retrieved	358	35	49	22	15	14	3	2	2	3	503
Proportion (%)	71.2%	7.0%	9.7%	4.4%	3.0%	2.8%	0.6%	0.4%	0.4%	0.6%	100.0%
Cumulative recovery rate (%)	59.6%	65.4%	73.5%	77.2%	79.7%	82.0%	82.5%	82.9%	83.2%	83.7%	83.7%

Binary logistic regression was used to evaluate the effect of the number of flushings on oocyte retrieval (Fig. 3). When the initial aspirate was set as the contrast, each subsequent flushing had a significant higher probability of retrieving more oocytes (*P* = 0.037 for Flush 1 and *P* = 0.000 for Flushes 2 to 9). When aspiration and 9 flushes was set as the contrast, Flush 0 (initial aspirate) to Flush 3 all had significantly lower RRs for the cumulative recovery rate (*P* < 0.01), while the RRs for 4 to 8 flushings were not significant (*P* > 0.05).

**Fig. 2:**
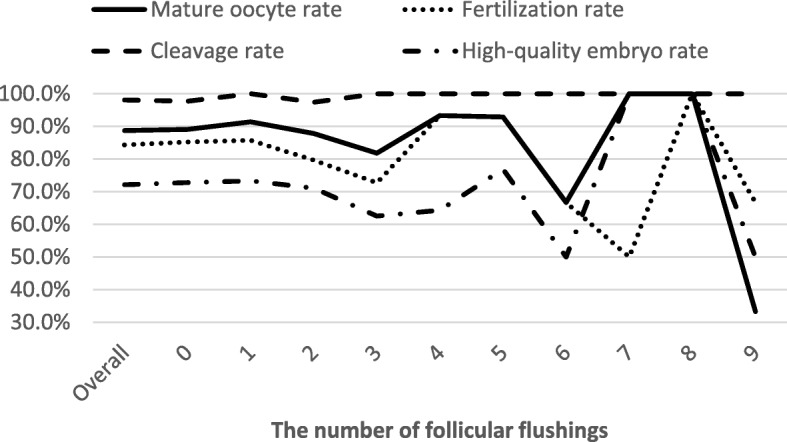
Laboratory embryology outcomes for each flushing. The mature oocyte rates, fertilization rates, cleavage rates, and high-quality embryo rates did not differ significantly among the flushing groups (*P*>0.05)

Figure 2 shows the quality of the oocytes and embryos obtained from the initial aspirate and subsequent flushes. Although a fluctuation existed, especially with higher numbers of flushings, the mature oocyte rates, fertilization rates, cleavage rates, and high-quality embryo rates did not differ significantly among the flushing groups (*P*>0.05). Furthermore, the IVF cycles were divided into three groups: (1) the initial aspirate group, (2) the subsequent flushes group, and (3) the aspirate and all flushes group, and the embryological outcomes were compared. The rates of mature oocytes, fertilization, cleavage and high-quality embryos were still comparable (*P* > 0.05; Table 4).

**Table 4:** Laboratory embryology outcomes for the aspirate and flushing groups

	Initial aspirate	Subsequent flushes	Aspirate & all flushes	*P*-value
Mature oocyte rate	89.1%(319/358)	87.6%(127/145)	88.7%(446/503)	0.888
Fertilization rate	85.2%(305/358)	82.1%(119/145)	84.3%(424/503)	0.683
Cleavage rate	97.7%(298/305)	99.2%(118/119)	98.1%(416/424)	0.613
High-quality embryo rate	72.8%(217/298)	70.3%(83/118)	72.1%(300/416)	0.879

**Fig. 3:**
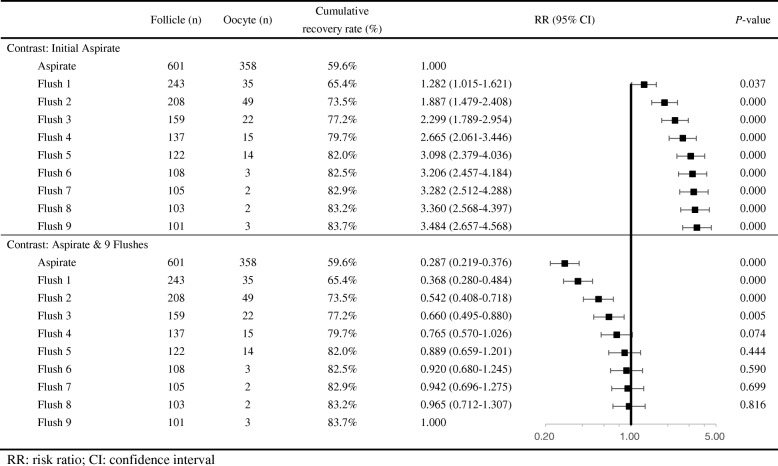
Risk ratios and 95% confidence intervals for the cumulative oocyte yield of each flush compared with the initial aspirate and 9 flushes. RR: risk ratio; CI: confidence interval

## Discussion

Although the evidence supporting the advantage of follicular flushing over direct aspiration is limited, the potential for recovering more oocytes and subsequently improving pregnancy outcomes remains, especially in POR patients and those undergoing natural cycle and minimal stimulation IVF [17, 18]. In clinical practice, many ART centers still perform follicular flushing as a routine oocyte retrieval procedure [6].

Both clinicians and patients are confused regarding whether follicular flushing is really effective for increasing the number of oocytes. Given the possible adverse effects on embryo quality and pregnancy outcomes [17, 21] and the inevitability of increasing operation time and anesthesia risks [16, 18], this procedure may have no clinical value if more oocytes cannot be recovered with recurrent flushing. In addition, the oocyte recovery rate can hardly reach 100%. When no oocyte has been retrieved, what is the optimal point at which to stop follicular flushing to avoid wasting time and human resources?

We conducted this study to answer these questions. We found a cumulative recovery rate of 83.7% and a mean oocyte retrieval of 1.73 ± 0.96 per cycle with 9 flushes, significantly higher than the 59.6% and 1.23 ± 1.00, respectively, obtained with direct aspiration. An obvious ascending tendency in the cumulative recovery rate was observed before the 4th flushing; however, after 4 flushings, the cumulative rate did not significantly increase. Our study also showed that follicular flushing had no detrimental effects on oocytes and embryos.

To date, few studies have investigated the efficacy of follicular flushing in POR patients, and the results were conflicting. Studies favoring flushing in POR patients found that subsequent flushes were associated with increasing proportions of recovered oocytes, advanced embryo morphology, and increases in the implantation and clinical pregnancy rates [12, 22]. However, Mendez Lozano D, et al. [12] only studied patients with a single dominant follicle, and Souza A, et al. [22] had a relatively unsatisfactory cumulative oocyte recovery rate (76.8%) due to the use of lower aspirate pressure (100 mmHg). Our study included POR patients with 1–3 dominant follicles because they were more representative than those with a single follicle and could potentially benefit more from follicular flushing than patients with 4 or more mature follicles. Our aspirate pressure was higher (140 mmHg), as was our recovery rate (83.7%). Nonetheless, our study showed analogous outcomes.

Studies against flushing in POR patients have reported no significant differences between direct aspiration and follicular flushing in the number of retrieved oocytes, fertilization rates, or embryo quality [13, 21, 23, 24]. In addition, the pregnancy outcomes of follicular flushing were not improved; rather, they were compromised [21]. However, the designs and results of these studies were not flawless. Levens E, et al. [13] excluded women with < 4 follicles and therefore probably did not represent very poor responders. The diameters of the follicles aspirated in 3 RCTs [13, 21, 24] were ≥ 12 mm or ≥ 10 mm, which is too wide a range to evaluate the presence of mature follicles that can yield mature oocytes. Mok-Lin E, et al. [21] and von Horn K, et al. [24] designed their RCTs on a scale that would detect a one-oocyte difference between the aspiration and flushing groups. In our study, the mean number of oocytes retrieved was approximately 0.5 more in the aspiration and flushing group than in the direct aspiration group. To obtain such a minimal difference, the scales of RCTs should be much larger.

Another reason the efficacy of follicular flushing remains controversial is that too many factors can impact the procedure, and inevitably, researchers in different clinics cannot perform oocyte retrieval the same way. The difficulty of aspiration (based on the proficiency of the operator), the inner diameter and length of the needle, aspiration pressure, follicle diameter, volume of flush fluid, number of flushes, and the intra-follicular pressure caused by the injection of flush fluid may all influence oocyte and embryo outcomes [18, 25]. In our study, the optimal number of flushings was four; however, other studies reported that 1 to 2 flushes could produce a commendable cumulative recovery rate [3, 26]. The wider inner needle diameter and the higher aspirate pressure used in their studies might account for this difference. Increased intra-follicular pressure and small-diameter follicles have been reported to have correlations with poor embryo quality and a decreased pregnancy rate [21, 25]. In further studies, including RCTs and non-randomized trails, researchers should attempt to standardize these influential factors to achieve a more reliable result.

Some studies have evaluated the effect of follicular flushing on pregnancy rates; however, we selected the number of oocytes and not pregnancy outcomes as our primary outcome because most of the POR patients received a two-embryo transfer, and the majority of patients who achieved pregnancy had only one fetus. Because in the routine procedure, the embryo derived from direct aspiration was often transferred along with one derived from follicular flushing, it is difficult to determine which one was implanted. What is easy to overlook is that in some RCTs, embryos transferred in the follicular flushing group could also have been derived from the initial aspiration. Even if the purpose was to evaluate the effect of more oocytes on pregnancy, the cumulative pregnancy rate of all viable embryos transferred should be evaluated rather than the pregnancy rate associated with a single transfer procedure. To obtain a more convincing conclusion, studies should be designed to preform single embryo transfer or the transfer of two embryos derived from the same procedure (either direct aspiration or follicular flushing).

## Conclusions

We suggest that follicular flushing may increase the number of oocytes retrieved in POR patients undergoing IVF treatment without adverse effects on oocyte or embryo quality. Four follicular flushings may be an optimal number to achieve the balance between maximizing the number of oocytes and avoiding a waste of time and human resources. Our study was limited by its retrospective design and the lack of a control group. Additional well-designed RCTs are needed to confirm the effectiveness of this procedure.

## Abbreviations

ART: assisted reproductive technology
CI: confidence interval
IVF: in vitro fertilization
OCCC: oocyte-corona-cumulus complex
POR: poor ovarian response
RCT: randomized controlled trial
RR: risk ratio
